# Supplementary material for: Biomimetic fibrous semiconducting micromesh via tuning phase separation for high-performance stretchable optoelectronic synapses
Source: Nat Commun. 2025 Sep 26;16:8483. doi: 10.1038/s41467-025-63430-1 (PMC12475285; doi:10.1038/s41467-025-63430-1)
Supplement: Supplementary file 1 — Supplementary information [file 41467_2025_63430_MOESM1_ESM.pdf]

Supplementary Information

Biomimetic fibrous semiconducting micromesh for high-performance stretchable optoelectronic synapses

Qing Zhou[1], *Xinzhao Xu*[1], *Gezhou Zhu*[1], *Wenhao Li*[1], *Haoqing Zhang*[1], *Lin Shao*[1], *Zhihui Wang*[2]*, *Yunqi Liu*[1]*, and *Yan Zhao*[1,2]*

[1]*Laboratory of Molecular Materials and Devices, Department of Materials Science Fudan University, Shanghai 200433, P.R. China*
[2]*Department of Respiratory and Critical Care Medicine, Changhai Hospital, Naval Medical University, Shanghai 200433, China*
E-mail: zhw_ecust@163.com, liuyq@fudan.edu.cn, zhaoy@fudan.edu.cn

Supplementary Figures and Tables

Section 1. Characterization of the neat N2200 conjugated polymer and semicrystalline elastomer PBAT compared with SEBS.

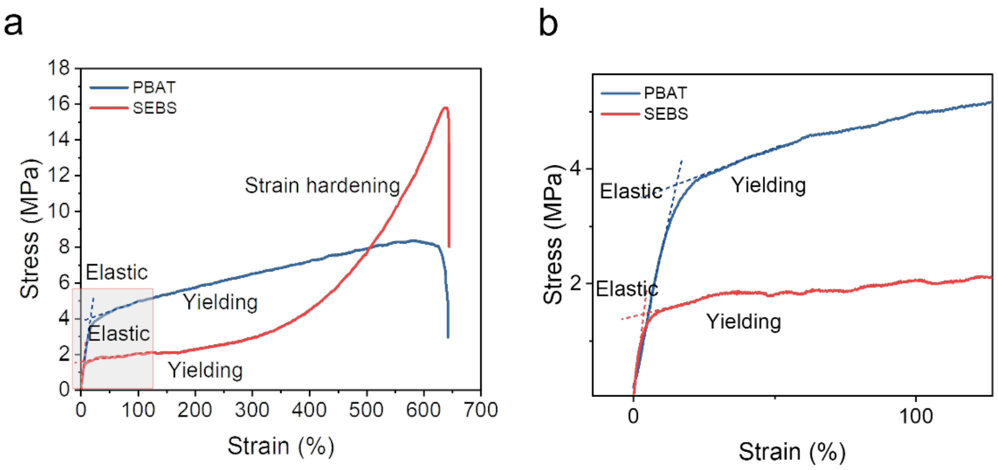

Supplementary Figure 1. Stress-strain curves of PBAT and SEBS (H1052).

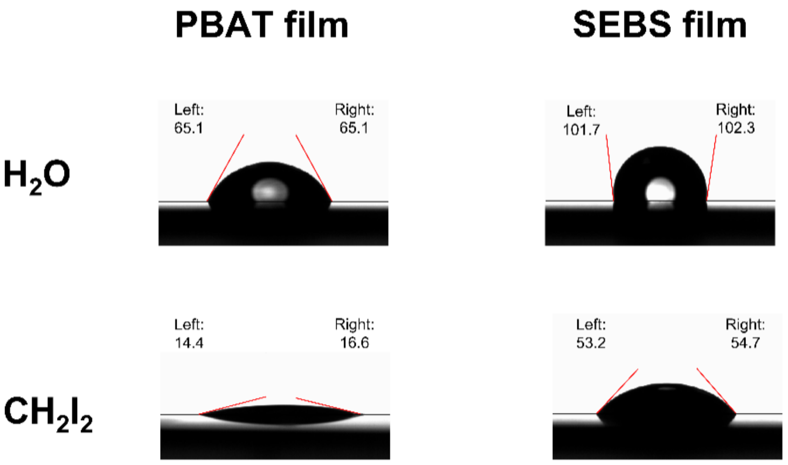

Supplementary Figure 2. Optical images of the snapshotted droplet showing the contact angles of the PBAT films and SEBS films.

Supplementary Table 1. Surface energies of the PBAT films and SEBS films.

materials	Contact angle (°)		Surface energy (mJ m^{-2})		
	H_2O	CH_2I_2	γ_S^d	γ_S^p	γ_S
PBAT	65.2	15.4	43.9	8.4	52.3
SEBS	102	54	32.2	0.1	32.3

Each contact angle was the average of three individual measurements.

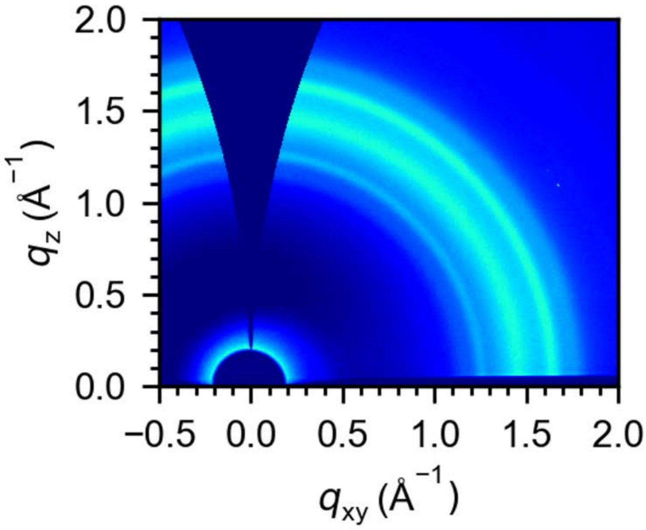

Supplementary Figure 3. Grazing incidence wide angle X-ray scattering (GIWAXS) pattern of PBAT.

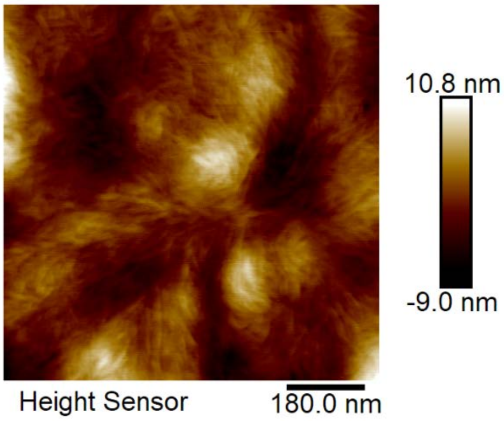

Supplementary Figure 4. Atomic force microscope (AFM) of PBAT film, Ra=2.2 nm.

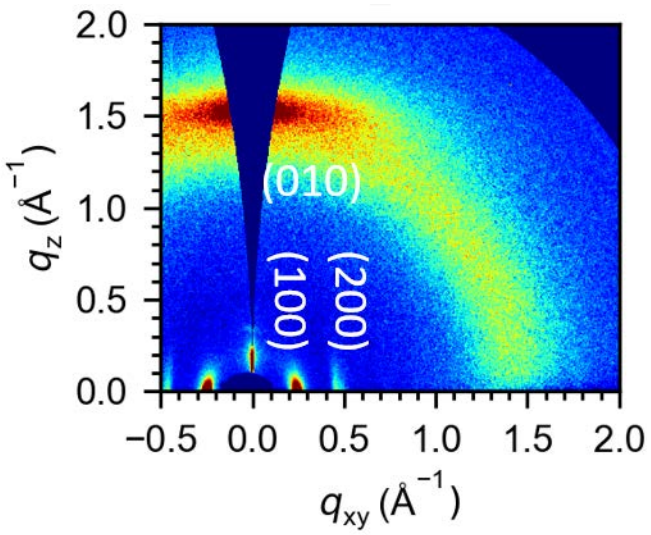

Supplementary Figure 5. GIWAXS pattern of neat film of N2200.

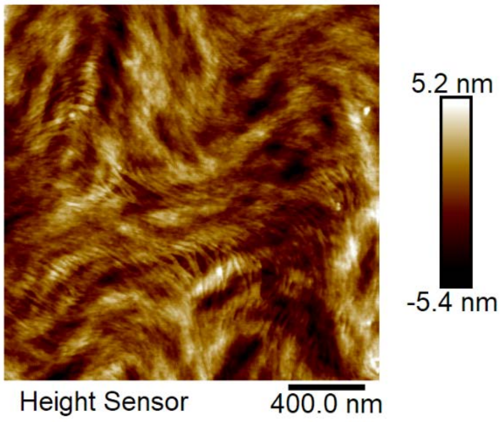

Supplementary Figure 6. The topography atomic force microscope (AFM) of N2200, Ra=1.8 nm.

Section 2. Pattern evolution in the OSC-insulator hybrid films.

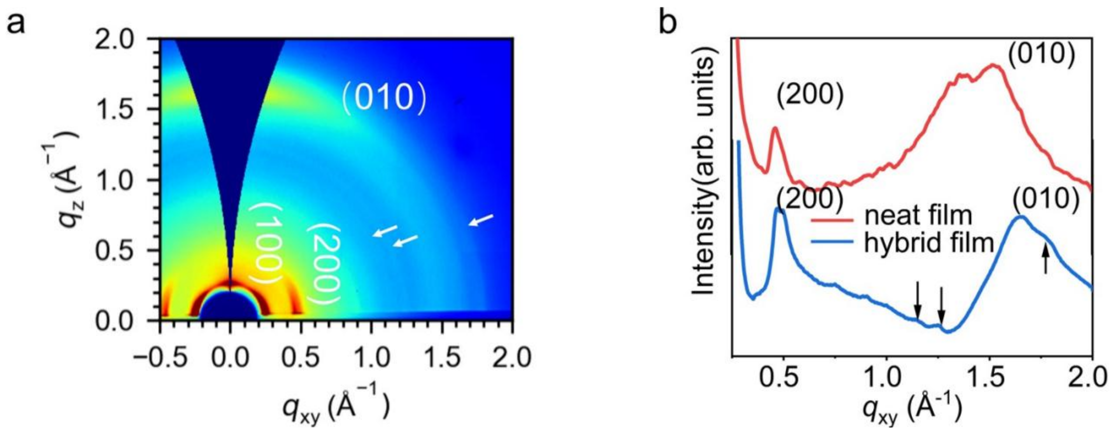

Supplementary Figure 7. (a) GIWAXS pattern of the FMM film of N2200. The white arrows marked the characteristic signals of the semicrystalline elastomer PBAT. (b) Profile of GIWAXS patterns of the FMM film and neat film of N2200.

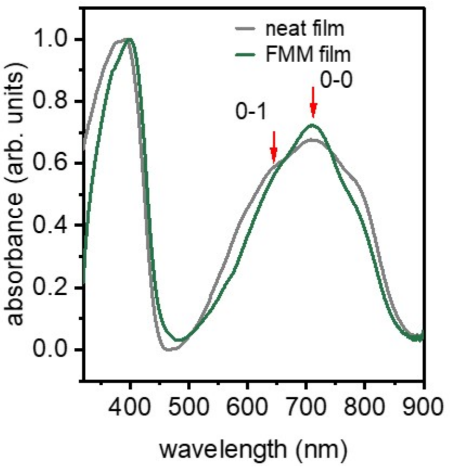

Supplementary Figure 8. UV-visible absorption spectrum of neat film and fibrous micromesh film (FMM film) of N2200. The stronger absorption at the 0-0 peak near 709 nm indicates enhanced chains aggregation in the FMM film.

Supplementary Table 2. Topography- and adhesion atomic force microscope (AFM) maps of hybrid films obtained under varying post-annealing treatments. PABT content ratio is 10 wt.% and 20 wt.%.

PBAT contents	As cast	120 °C	180 °C
10%	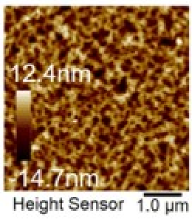	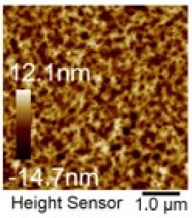	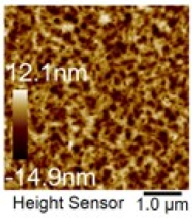
	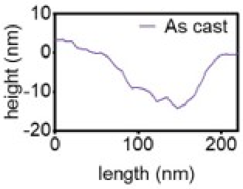	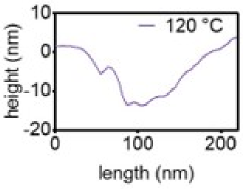	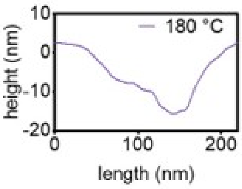
	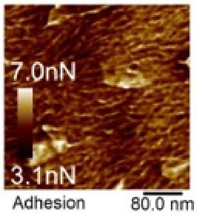	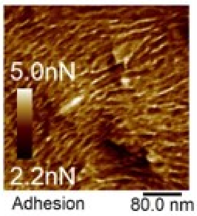	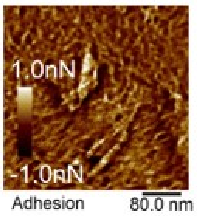
20%	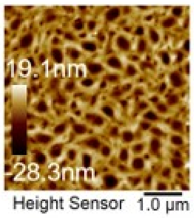	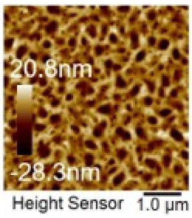	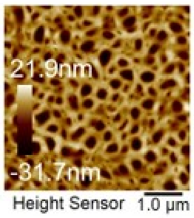
	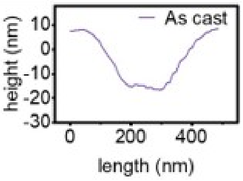	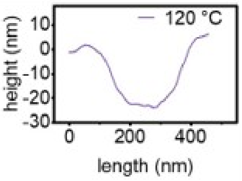	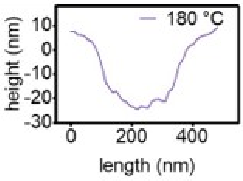
	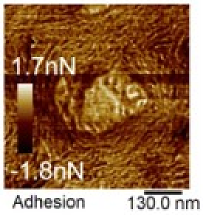	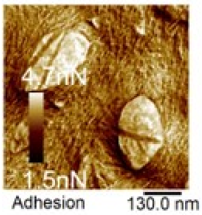	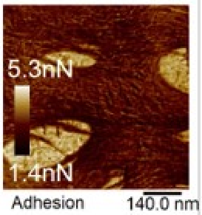

Supplementary Table 3. Topography- and adhesion atomic force microscope (AFM) maps of hybrid films obtained under varying post-annealing treatments. PABT content ratio is 40 wt.%.

<table>
<tr><th>PBAT content</th><th>As cast</th><th>120 °C</th><th>180 °C</th></tr>
<tr><td rowspan="3">40%</td>
<td>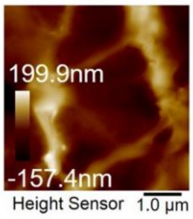
</td>
<td>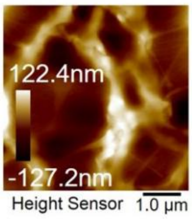
</td>
<td>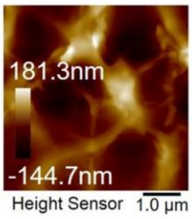
</td></tr>
<tr>
<td>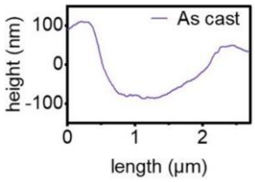
</td>
<td>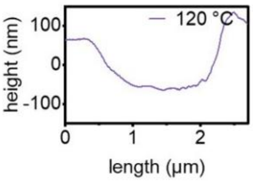
</td>
<td>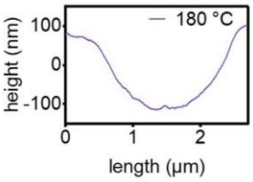
</td></tr>
<tr>
<td>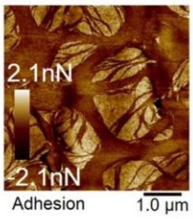
</td>
<td>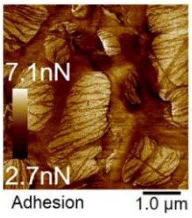
</td>
<td>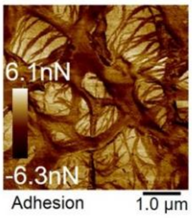
</td></tr>
</table>

Section 3. Electrical performance and mechanical stretchability of the OSC-insulator hybrid films with various contents ratios.

Electrical characterization

To evaluate the electrical performance of the investigated conjugated polymer films on silicon wafers, bottom-gate-top-contact field effect transistors were fabricated by directly spin-coating semiconducting films on top of OTS-modified 300-nm-SiO_2/Si, then Au (30 nm) was evaporated as the source and drain electrodes. The channel length (L) and width (W) are 30/50 μm and 1500 μm, respectively. All the operation above was carried out in the glove box. The electrical performance of transistors was measured with Keithley-4200 in the glove box. The field-effect hole mobility μ was calculated in the saturation regime of transistor operation from the equation:

$$\mu_{\mathrm{sat}} = (\frac{\partial\sqrt{I_{\mathrm{DS}}}}{\partial\sqrt{V_{\mathrm{GS}}}})^2 * (\frac{2L}{WC_i}) \quad (1)$$

where μ_{sat} is the saturation mobility; where I_{DS} is the drain-source current, V_{GS} is the gate voltage, V_{DS} is the drain-source voltage, C_i is the capacitance per unit area; L and W are the channel length and width, respectively; For both the OFETs on Si/SiO_2 substrates and stretchable substrates, we extracted the saturation mobilities from gate voltage region (V_{GS}=50~60 V) with a voltage range from 0V to 60 V. The mobilities was calculated with consideration of the device geometry and dielectric capacitance change.

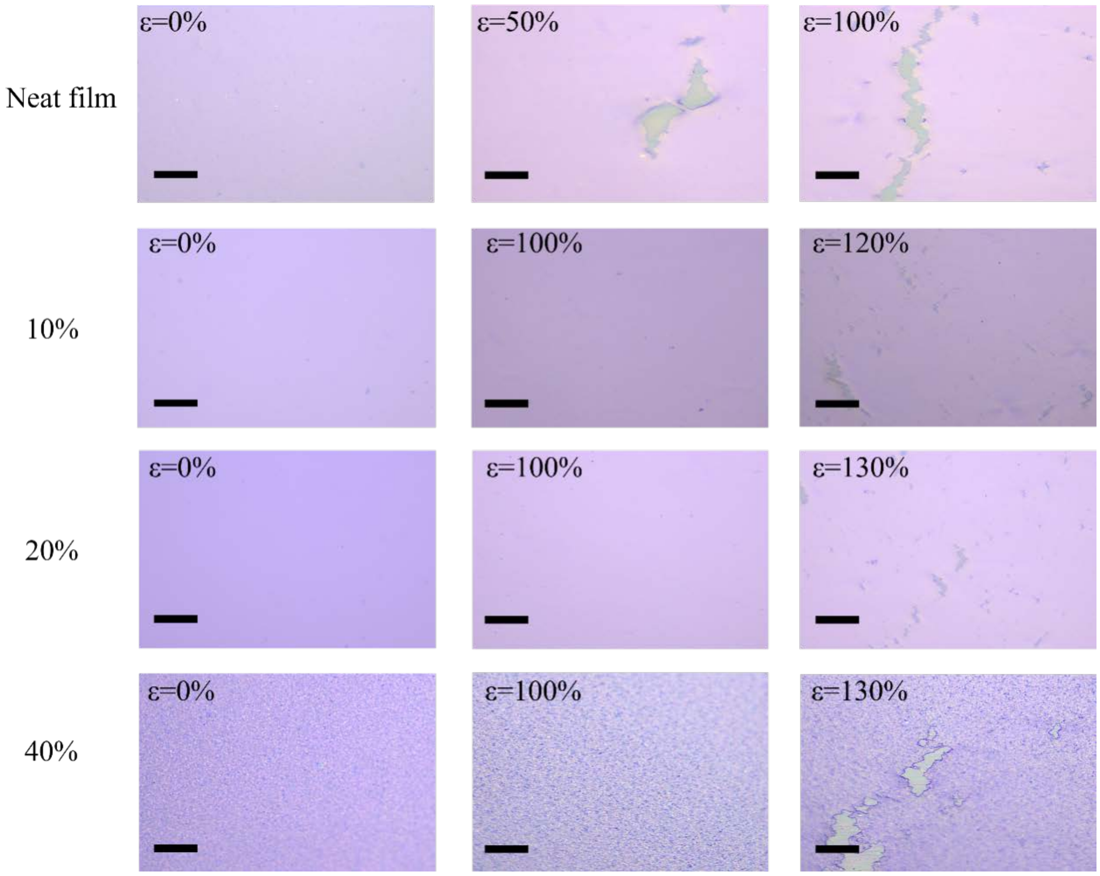

Supplementary Figure 9. Optical microscope images of the neat film and the hybrid polymer films with the insulator contents of 10%, 20% and 40%, respectively. The scale bar is 20 μm.

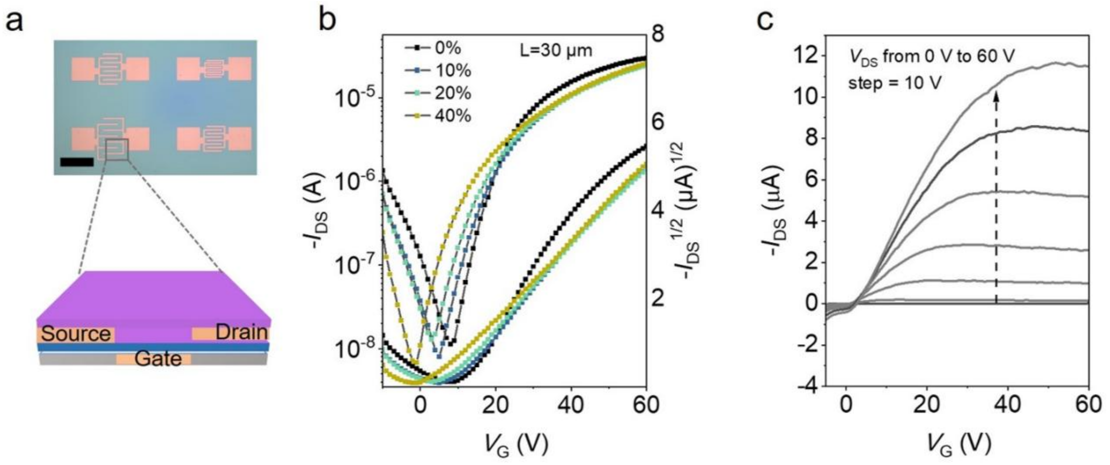

Supplementary Figure 10. Electrical performance of the hybrid films with controlled contents ratio. (a) Optical microscope image of the device on a Si/SiO_2 substrate with a bottom-gate-bottom-contact structure and the illustration of the device structure. Scale bar is 400 μm. (b) Representative transfer curves of the devices with controlled contents. (c) Output curve of the device with insulator content of 40% by weight (concentration of PBAT and N2200 were 3.3 mg ml^{-1} and 5mg ml^{-1}, respectively).

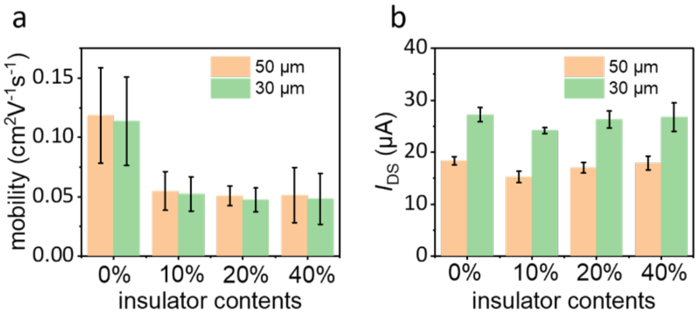

Supplementary Figure 11. Comparison of the a) mobility and b) on-current of the devices with different insulator contents. The channel length is 30 μm and 50 μm, respectively. Data are presented as mean values +/- SD, n=3. Data were obtained from 3 individual measurements.

Supplementary Table 4. Parameters of the device performance on Si/SiO_2 substrate.

PBAT Contents	Channel length(μm)	Mobility μ_{sat} ($cm^2V^{-1}s^{-1}$)	V_{th} (V)	I_{on}/I_{off}	I_{off} (nA)	Crack-on-set strain (%)
0%	30 μm	0.11	13.7	2.6E3	11.3	50
	50 μm	0.12	21.1	2.9E4	0.65	
10%[a]	30 μm	0.052	9.60	3.0E3	7.98	120
	50 μm	0.055	16.4	8.2E4	1.92	
20%	30 μm	0.047	7.5	2.0E3	12.7	130
	50 μm	0.051	7.2	1.3E5	1.36	
40%	30 μm	0.048	2.3	3.7E3	6.82	130
	50 μm	0.051	8.6	2.6E4	0.73	

[a] 10% represents the contents ratio of PBAT by weight.

Section 4. Phase separation in the OSC-insulator hybrid films.

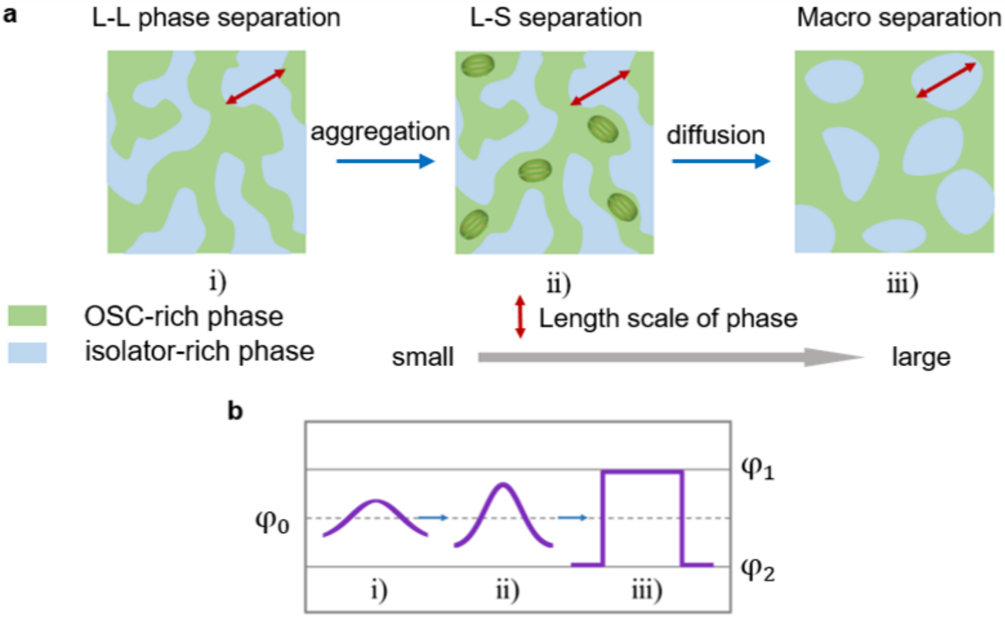

Supplementary Figure 12. Illustration of the phase separation of the binary polymer solution. (a) schematic of the pattern evolution of phase separation in the binary polymer solution. (b) Schematic of evolution of the phase composition. φ_0 represents the universal composition in the mixed solution, φ_1 and φ_2 represent the separated phase composition in the final hybrid film.

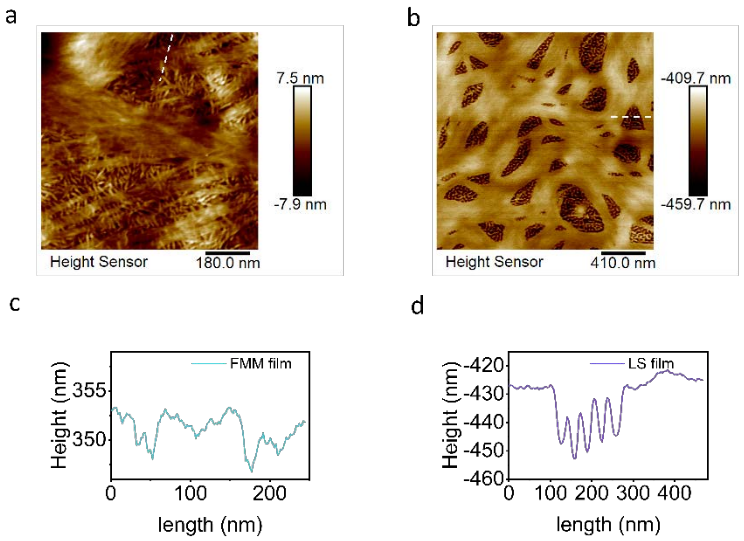

Supplementary Figure 13. The topography of the hybrid films. (a) Topography of FMM film. (b) Topography of LS film. (c) a line cut profile of the marked region in (a). (d) a line cut profile of the marked region in (b).The corresponding adhesion maps are listed in Figure 2b.

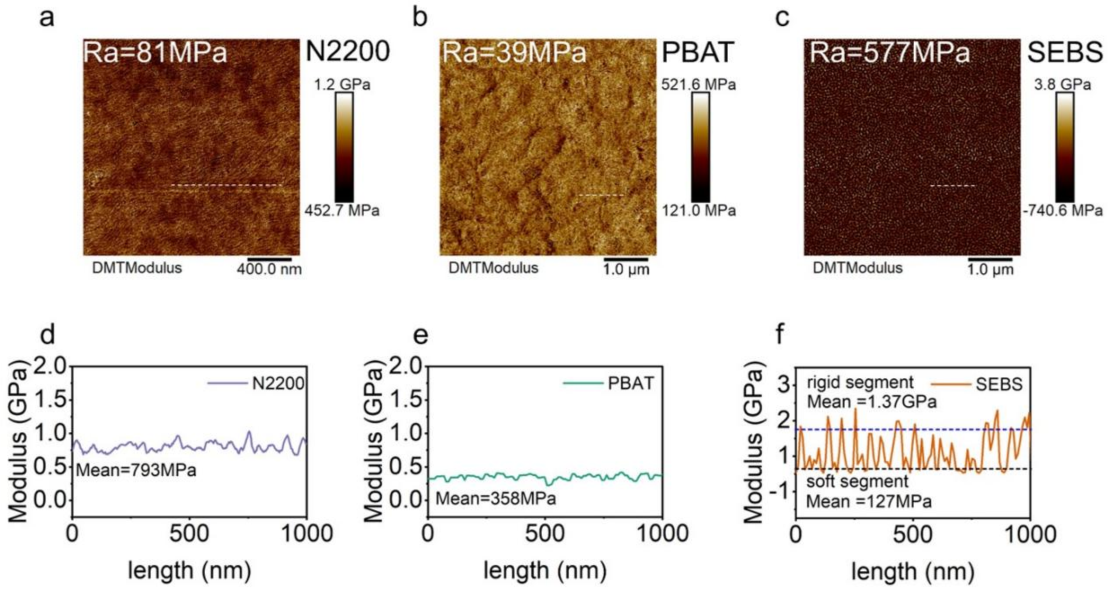

Supplementary Figure 14. The relative DMT modulus AFM of pristine N2200, PBAT, and SEBS. (a-c) modulus maps of N2200, PBAT, and SEBS. (d-f) profiles of the marked region in a-c, respectively. For the SEBS film, two separated DMT modules were obtained, corresponding to the micro phase separation of the rigid- and soft segment.

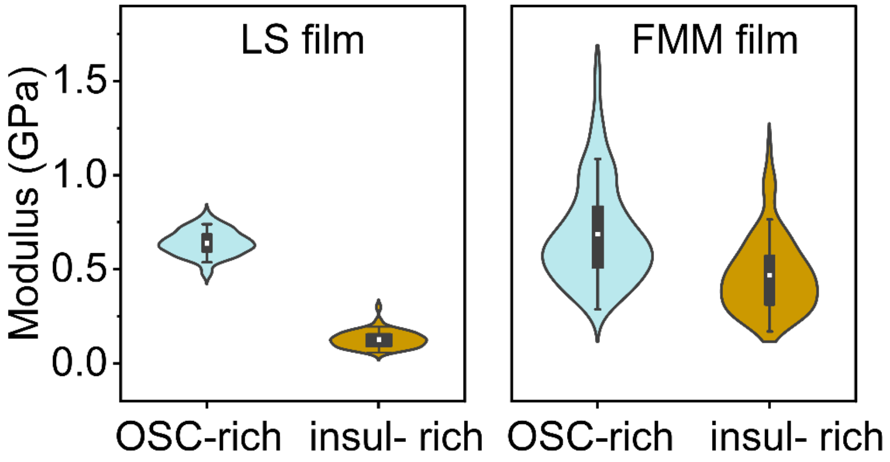

Supplementary Figure 15. Statistical results of the modulus mappings of LS film and FMM film, which are consistent with the adhesion mappings.

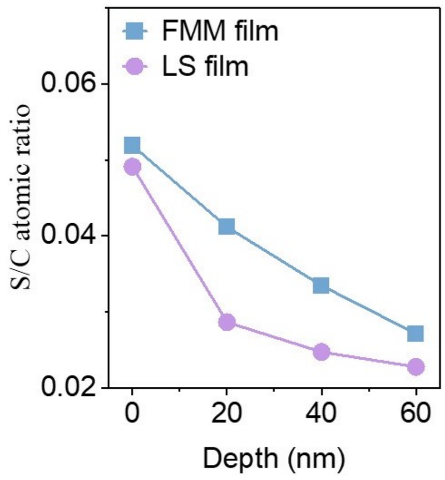

Supplementary Figure 16. Ratios between S2*p* peak and C1*s* peak (S/C ratio) from XPS spectra along the depth of the two hybrid films.

Section 5. The dependence of strain dissipation on pore diameter

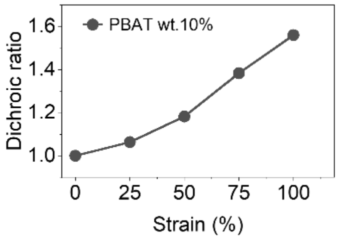

Supplementary Figure 17. Dichroic ratio of the hybrid films under different strains. The weight ratio of PBAT is 10%.

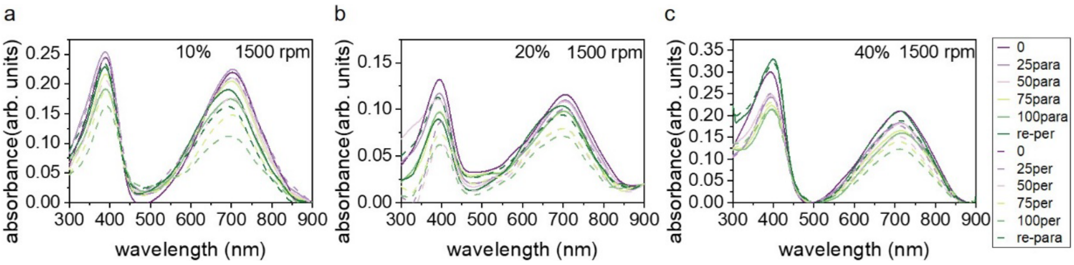

Supplementary Figure 18. Polarized UV-vis spectrum of the hybrid films with various contents ratio of insulator PBAT under varying strain. Solid- and dash lines represent the stretching direction parallel/perpendicular to the polarization, respectively**.**

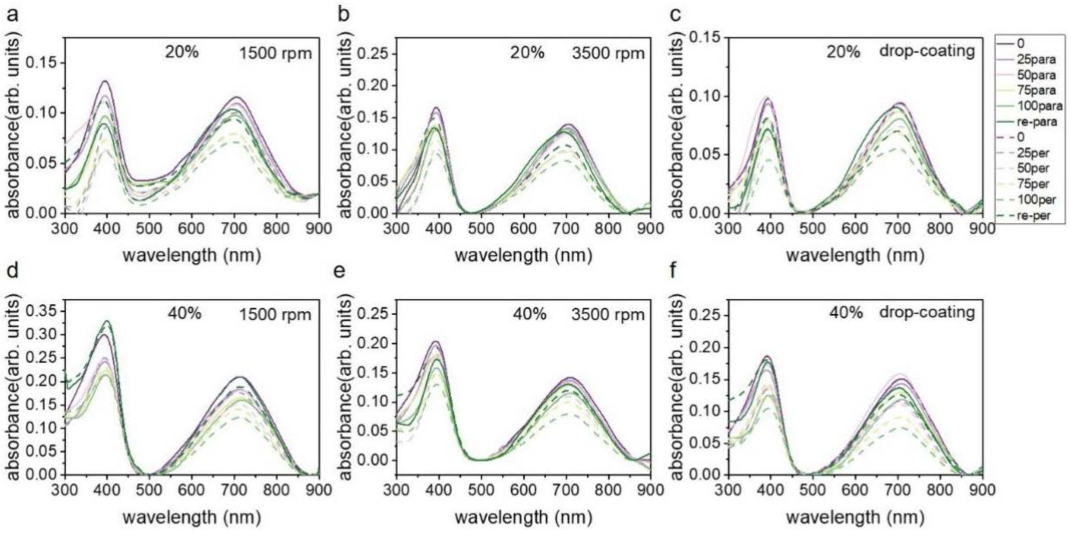

Supplementary Figure 19. Polarized UV-vis spectrum of hybrid films under varying strain. (a-c) Hybrid film (Contents ratio of PBAT is 20%) fabricated from spin-coating (speed=1500 rpm), spin-coating (speed=3500 rpm) and drop-coating methods, respectively. (d-f) Hybrid film (Contents ratio of PBAT is 40%) fabricated from spin-coating (speed=1500 rpm), spin-coating (speed=3500 rpm) and drop-coating methods, respectively. Solid- and dash lines represent the stretching direction parallel/perpendicular to the polarization, respectively.

Section 6. Morphology of the OSC films under strain.

Supplementary Table 5. Structural differences of neat film of N2200, FMM film and LS film*.

Structural difference		neat film	FMM film	LS film
Composition		N2200	N2200/PBAT (6:4)	N2200/SEBS (6:4)
Film morphology		Dense	Mesh structure	Mesh structure
		Non-fibrous	Fibrous	Non-fibrous
	SEM	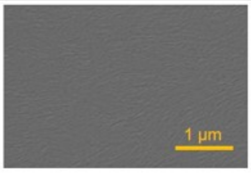	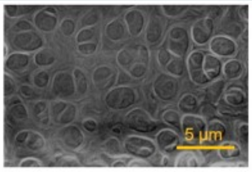	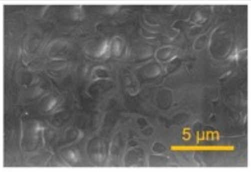
	adhesion	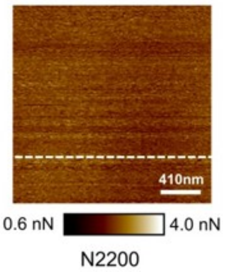	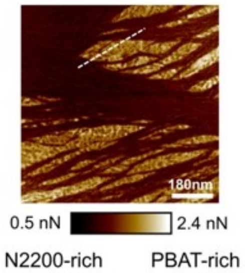	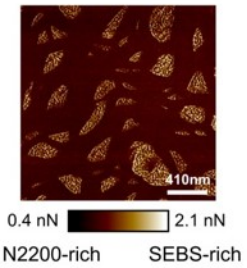
Phase composition		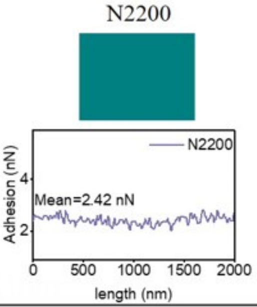	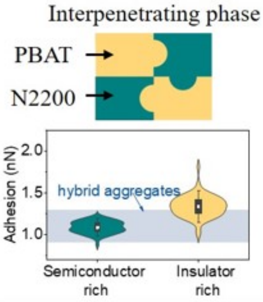	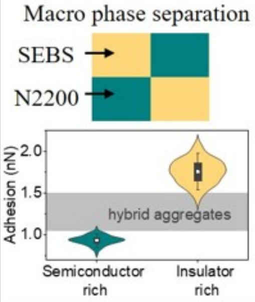

*For the FMM film and LS film, the conjugated polymer is N2200, and the insulator component is PBAT and SEBS, respectively.

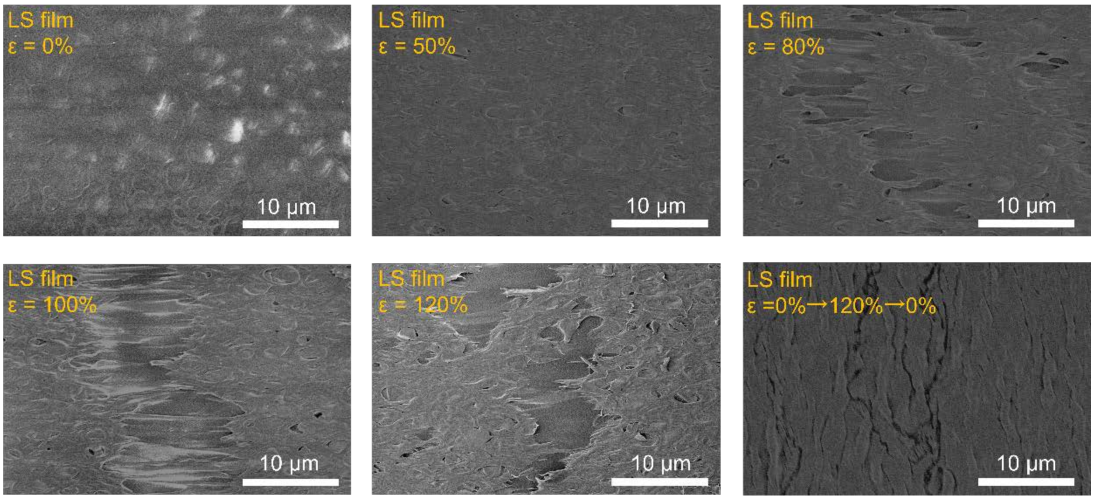

Supplementary Figure 20. SEM images of an LS film under various strains.

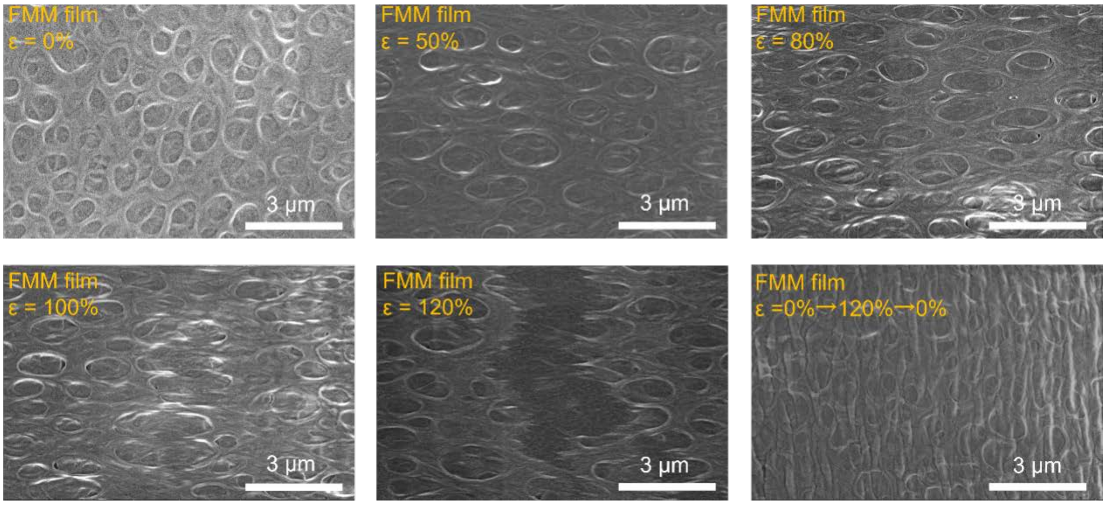

Supplementary Figure 21. SEM images of an FMM film under various strains.

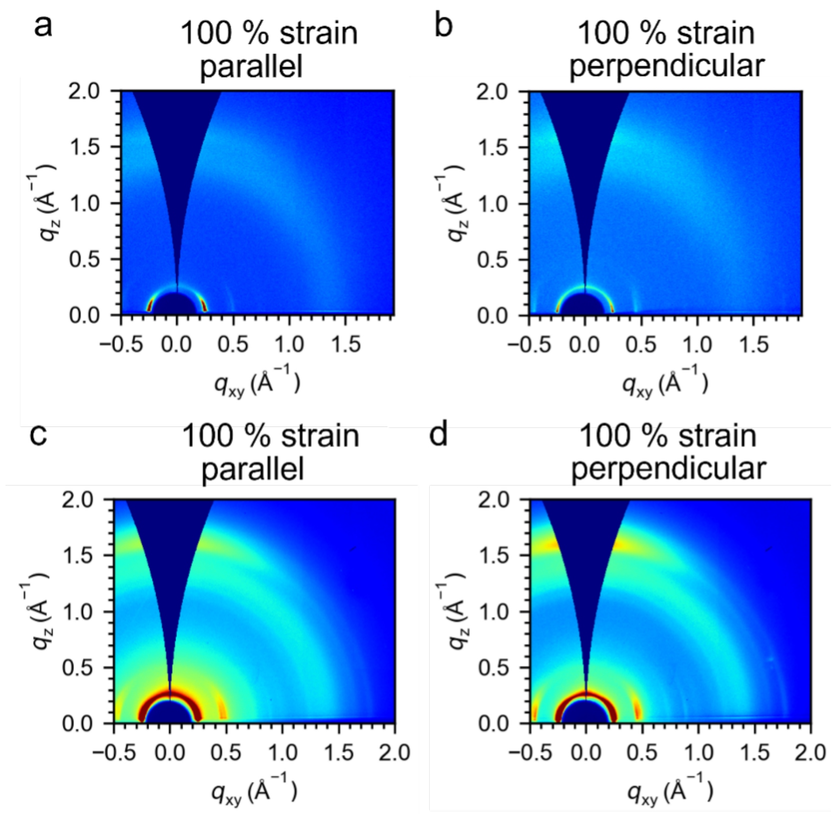

Supplementary Figure 22. GIWAXS patterns and the corresponding profiles of a, b) the neat film and c, d) the FMM film.

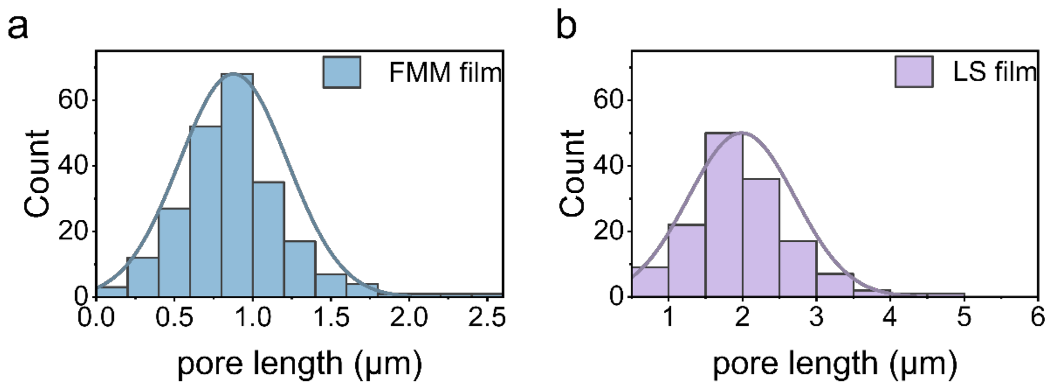

Supplementary Figure 23. Pore length distribution in the hybrid films.

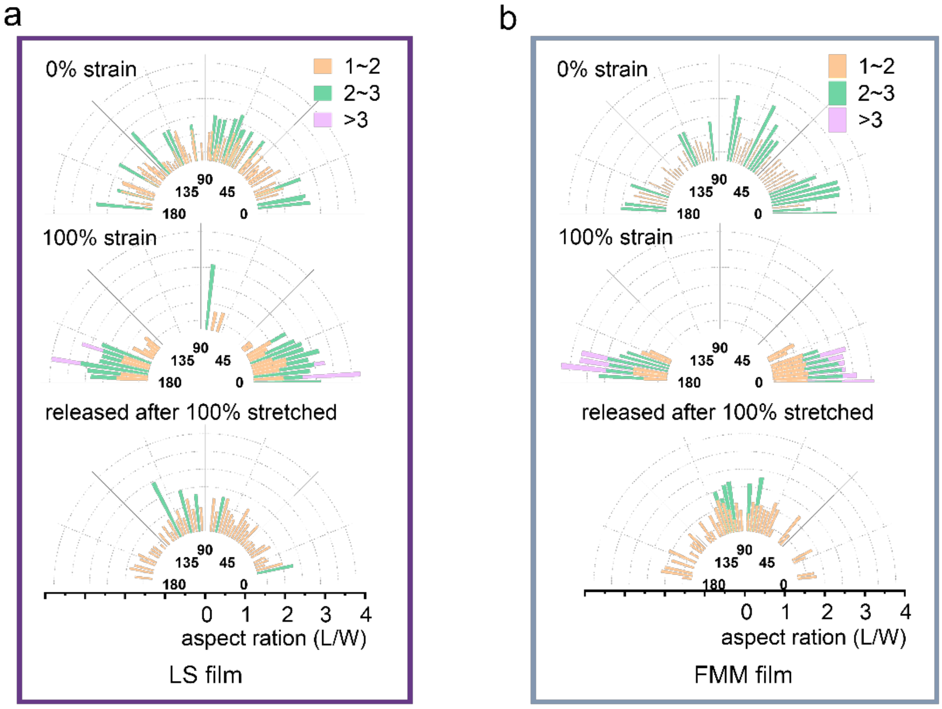

Supplementary Figure 24. Pore in-plane orientation distribution in the hybrid films before stretching and after release. Yellow panels represent pores with respect ratio lager than 1 but smaller than 2, green panels represent pores with respect ratio larger than 2 but smaller than 3, pink panels represent pores with respect ratio larger than 3. After releasing the strain, pores in the hybrid films exhibited orientation similar as the initial state, indicating the well-preserved microstructure under repeating strain.

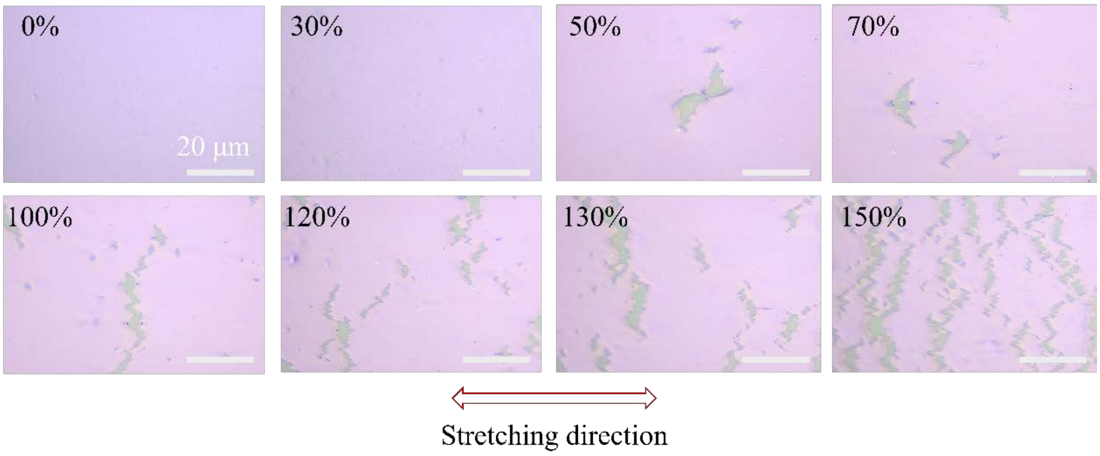

Supplementary Figure 25. Optical microscope images of neat N2200 film, with strain up to 150%, focusing on the topography before and after crack propagation.

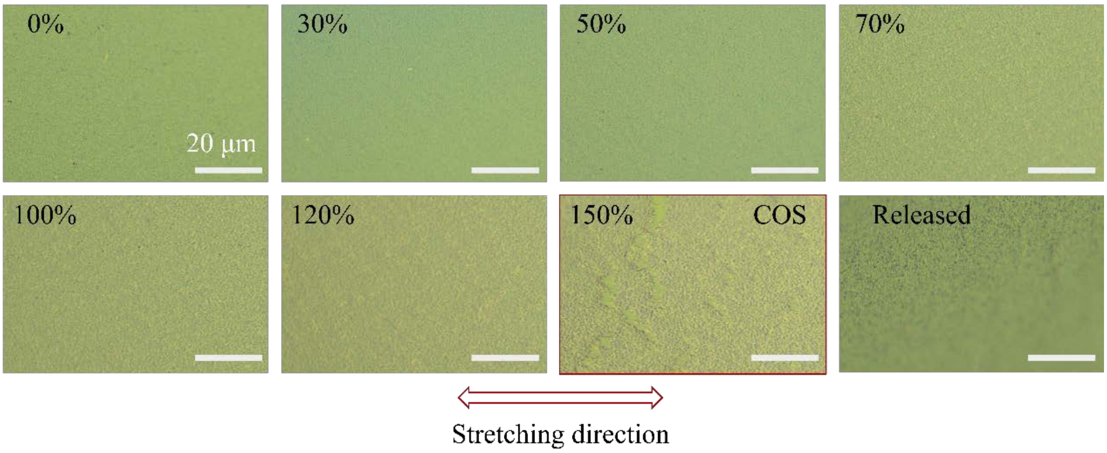

Supplementary Figure 26. Optical microscope images of LS film, with strain up to 150%, focusing on the topography before and after crack propagation.

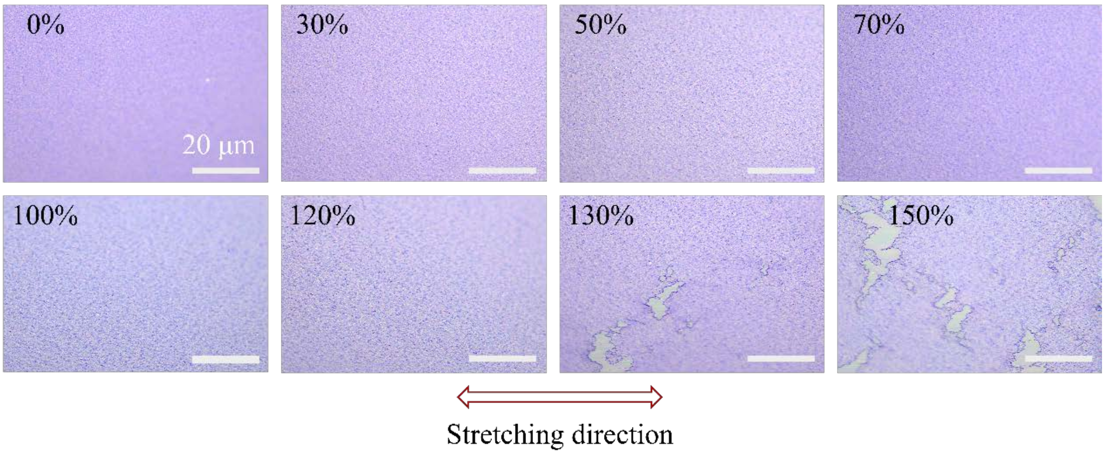

Supplementary Figure 27. Optical microscope images of FMM film, with strain up to 150%, focusing on the topography before and after crack propagation.

Section 7. Electrical performance of the fully stretchable OFETs.

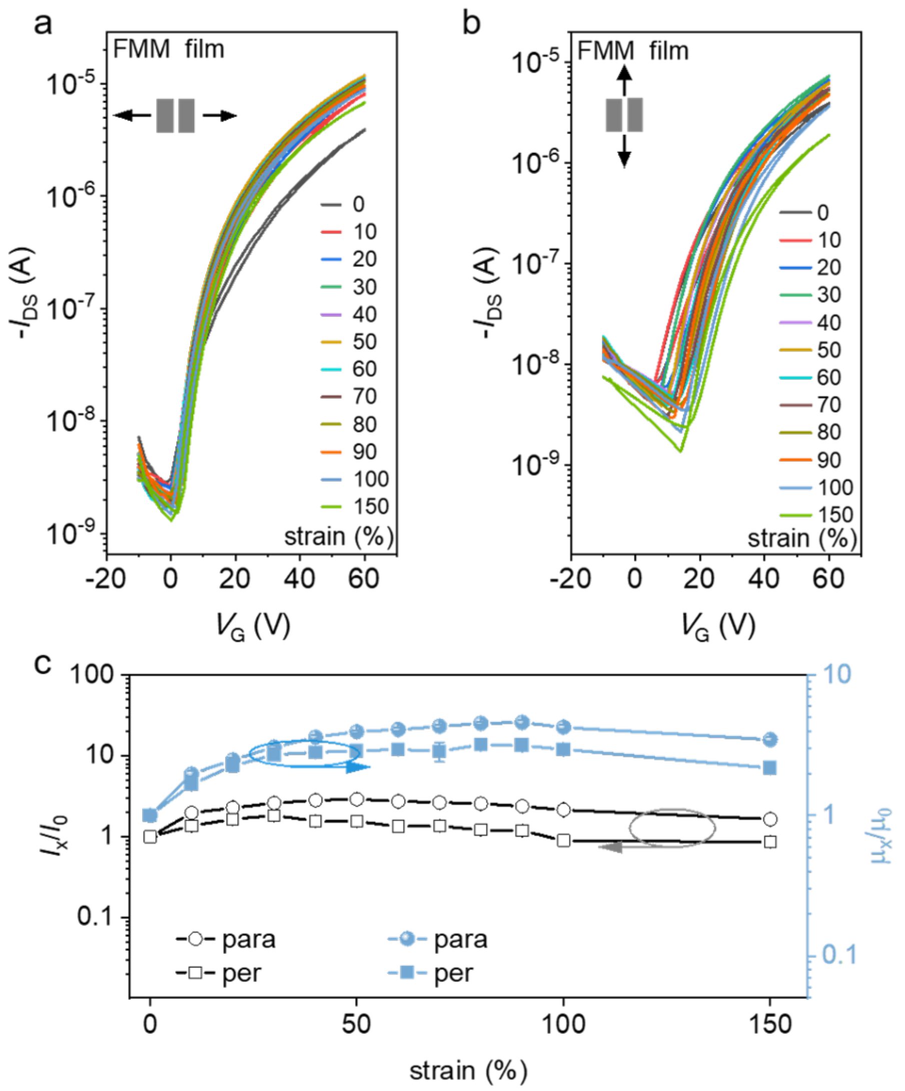

Supplementary Figure 28. Transfer curves of stretchable organic transistors under various strains employing FMM films. (a) Strain was parallel to the charge transport. (b) Strain was perpendicular to the charge transport. (c) On-current and the corresponding mobility of the fully stretchable OFETs employing FMM films of N2200 under various strain. I_x represents on-current in OFET under strain, I_0 represents on-current in OFET at initial state.

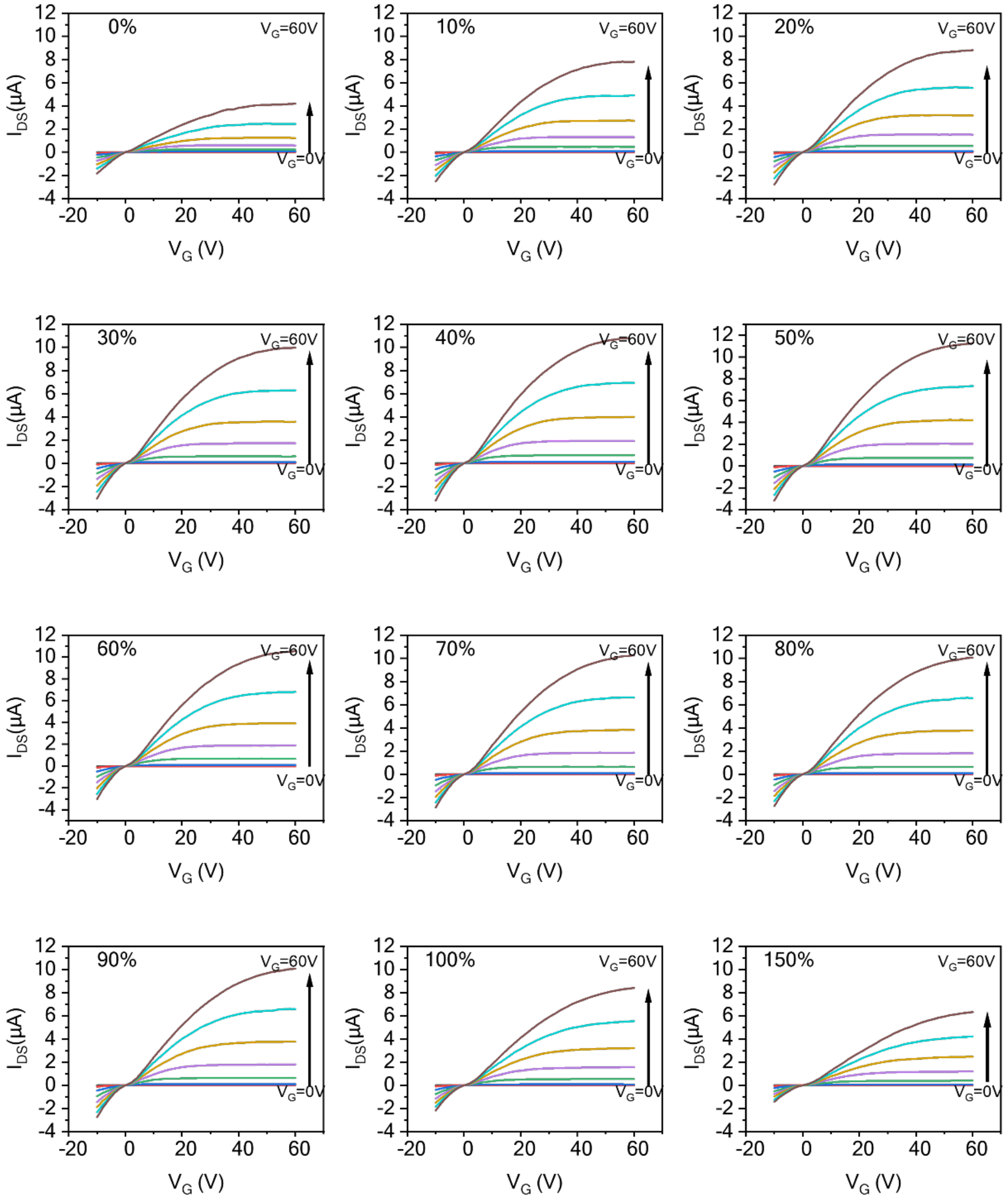

Supplementary Figure 29. Output curves of one stretchable organic transistor employing FMM films under various strains along the channel.

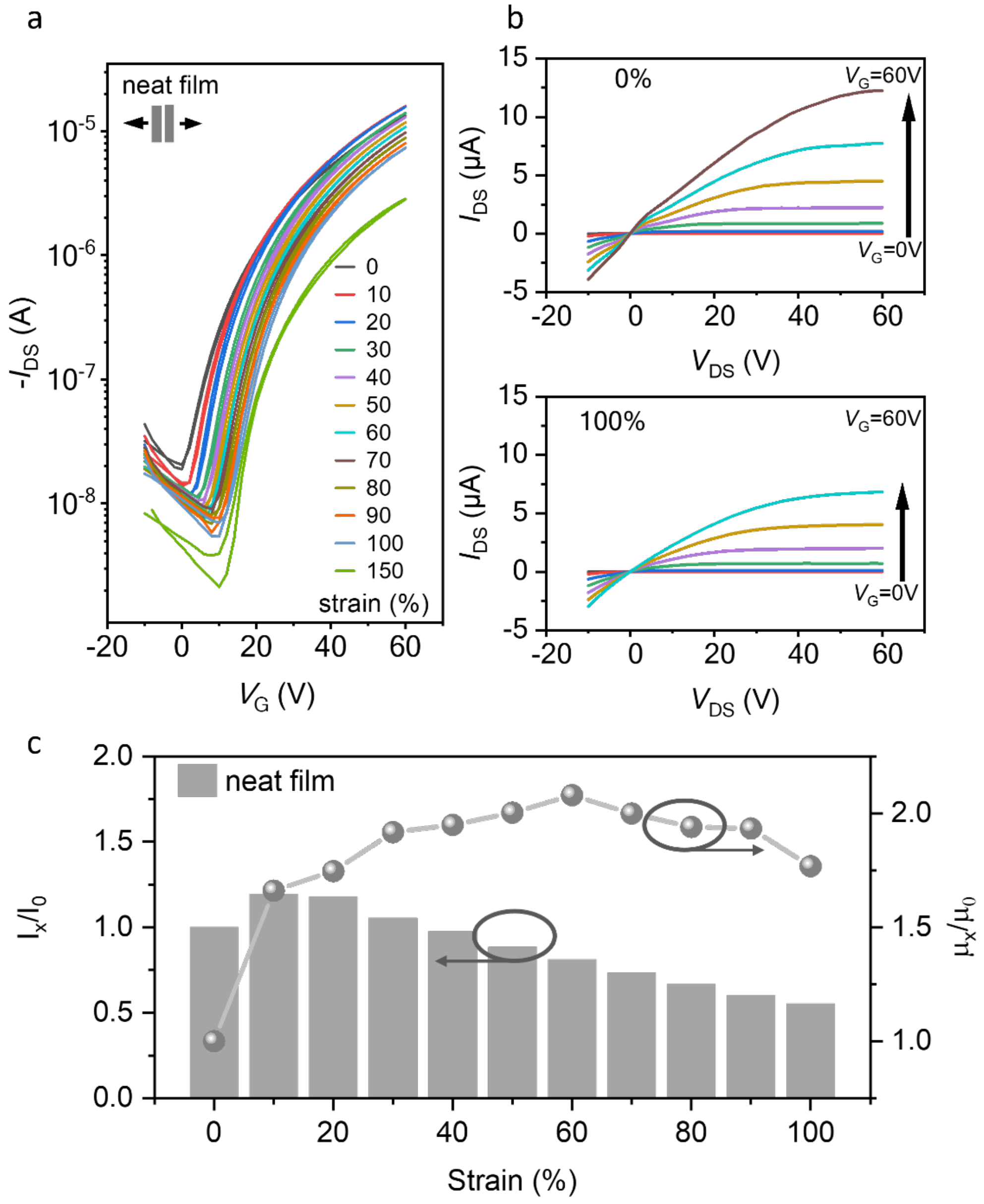

Supplementary Figure 30. Electrical performance of the stretchable organic transistors employing neat N2200 film under various strains parallel to the channel. (a) Transfer curves of one stretchable organic transistor. (b) Output curves of one stretchable organic transistor at initial state and under 100% strain. (c) The relative ratio of on-current and mobility of one stretchable organic transistor under various strains.

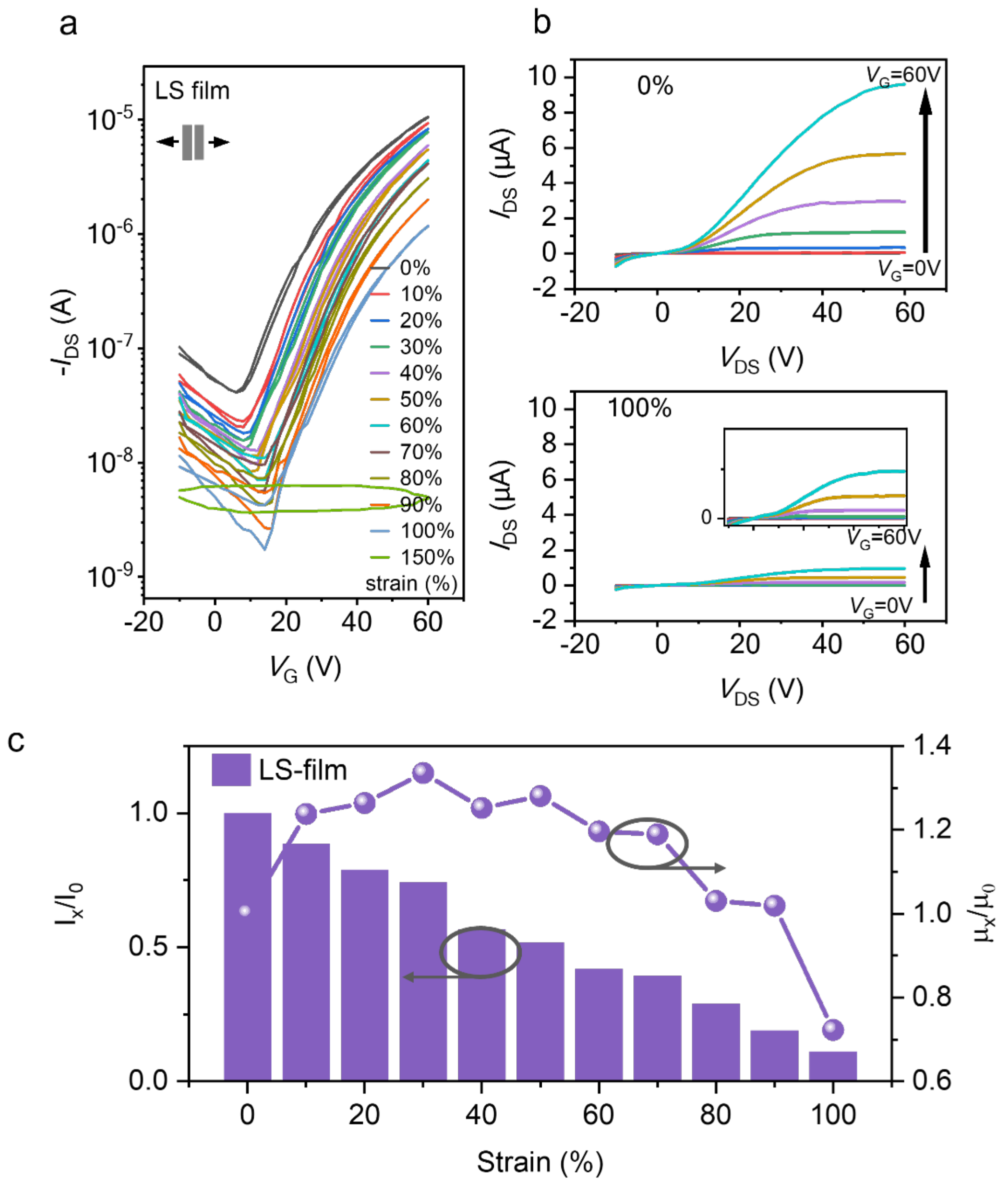

Supplementary Figure 31. Electrical performance of the stretchable organic transistors employing LS film under various strains parallel to the channel. (a) Transfer curves of one stretchable organic transistor. (b) Output curves of one stretchable organic transistor at the initial state and under 100% strain. (c) The relative ratio of on-current and mobility of one stretchable organic transistor under various strains.

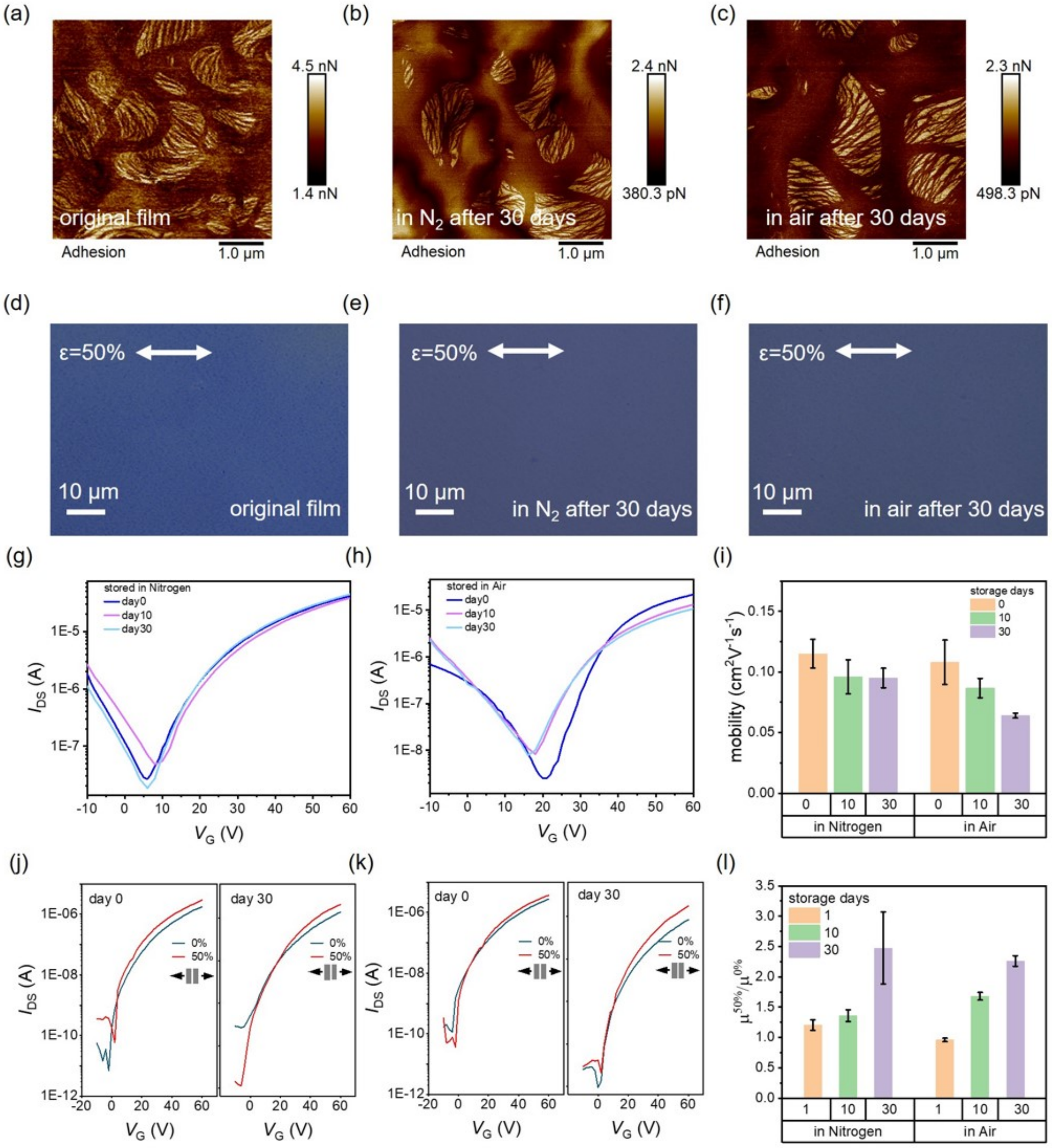

Supplementary Figure 32. Stability of film morphology and devices performance stored in glove box and humidity control cabinet. The adhesion images of FMM films of N2200 a) as annealed, b) stored in nitrogen for 30 days c) stored in air for 30 days. The optical microscope images of FMM films of N2200 under strain of 50% at different storage conditions d) the original film, e) in nitrogen after 10 days and f) in air after 10 days. The stretching direction is displayed by the arrows inserted. Transfer curves of OFETs on Si/SiO_2 based on FMM films of N2200 at different storage conditions g) in nitrogen and h) in air. (i) The corresponding mobility of devices in g) and h). Data are presented as mean values +/− SD, n=3.Transfer curves of stretchable OFETs based on FMM films of N2200 at different storage conditions j) in nitrogen and k) in air. (l) The corresponding mobility of devices in j) and k). Data are presented as mean values +/− SD, n=3. The temperature and humidity in glove box filled with nitrogen and humidity control cabinet is 21 °C and16 ppm and 25 °C and 46% RH.

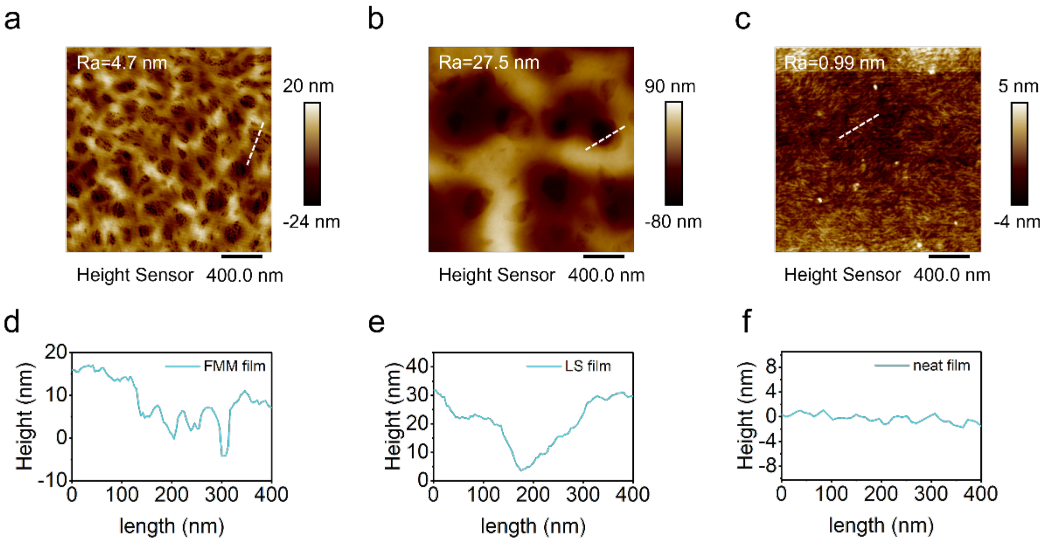

Supplementary Figure 33. The topography of hybrid films of DPPTT. (a) FMM film. (b) LS film. (c) neat film. and the height profile of marked region in the corresponding films. (d) FMM film. (e) LS film. (f) neat film.

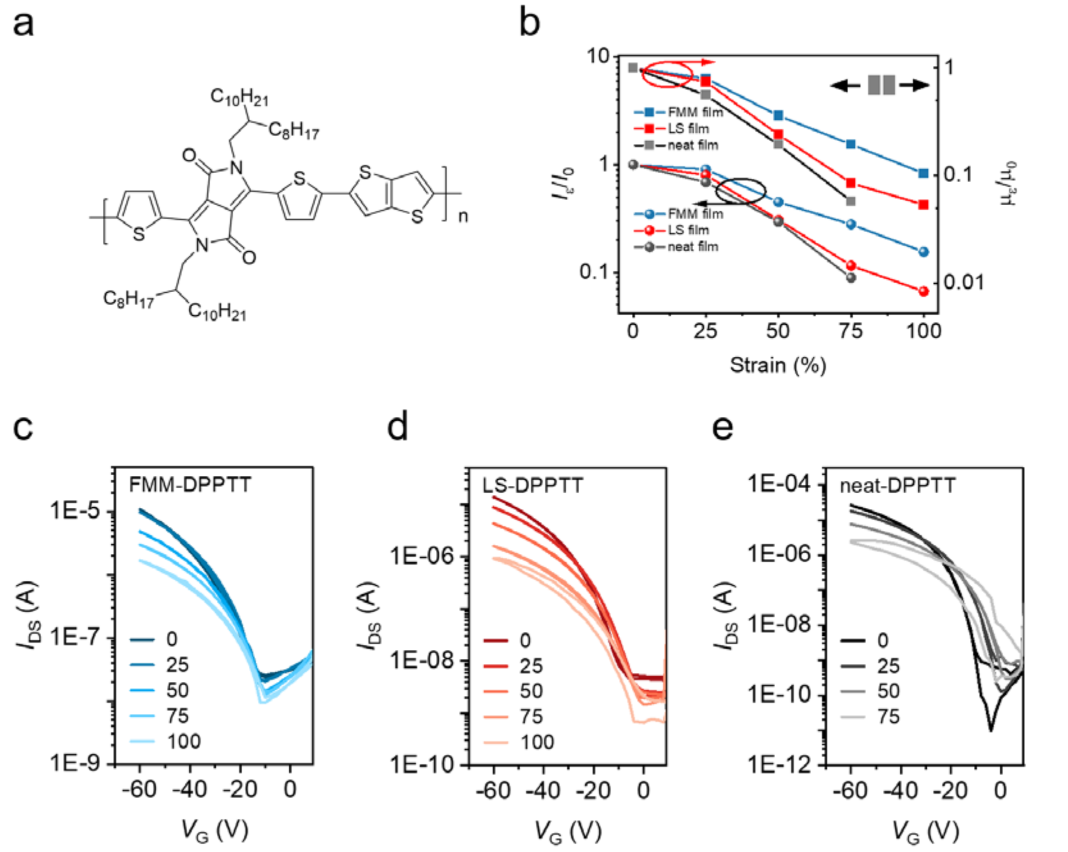

Supplementary Figure 34. Electrical performance of the stretchable organic transistors employing DPPTT hybrid films. (a) Chemical structure of the DPP-TT. (b) On-current and the corresponding mobility of the fully stretchable OFETs under various strain. I_ε represents on-current in OFET under strain, I_0 represents on-current in OFET at initial state. The strain is parallel to the tensile strain direction. The corresponding transfer curves are shown in c) FMM film, d) LS film, e) neat film.

Supplementary Table 6 The device geometry and dielectric capacitance in stretchable transistors under various strains

Strain (%)	Stretching parallel to channel		Stretching perpendicular to channel		Capacitance (nF cm^{-2})
	Length (μm)	Width (μm)	Length (μm)	Width (μm)	
0	100	5000	5000	100	1.029
10	110	4900	5500	98	1.097
20	120	4800	6000	96	1.169
30	130	4700	6500	94	1.238
40	140	4600	7000	92	1.266
50	150	4500	7500	90	1.311
60	160	4400	8000	88	1.348
70	170	4300	8500	86	1.379
80	180	4200	9000	84	1.401
90	190	4100	9500	82	1.421
100	200	4000	10000	80	1.420

All the channel widths under strain were calculated according to the Poisson's ratio of PDMS substrates (ν= 0.5) and verified by optical microscope.

Section 8. Synaptic phototransistors employing the FMM film of DPPTT.

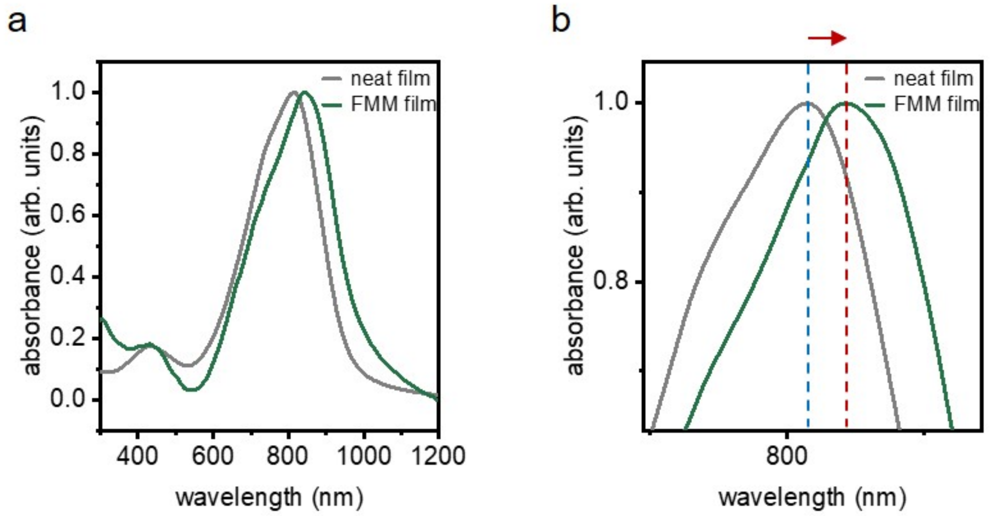

Supplementary Figure 35. UV-visible absorption spectrum of neat film and FMM film of DPPTT. The red shift of the absorption peak indicates the enhanced aggregation of polymer chains in the FMM film, as a result of the co-assembly effect.

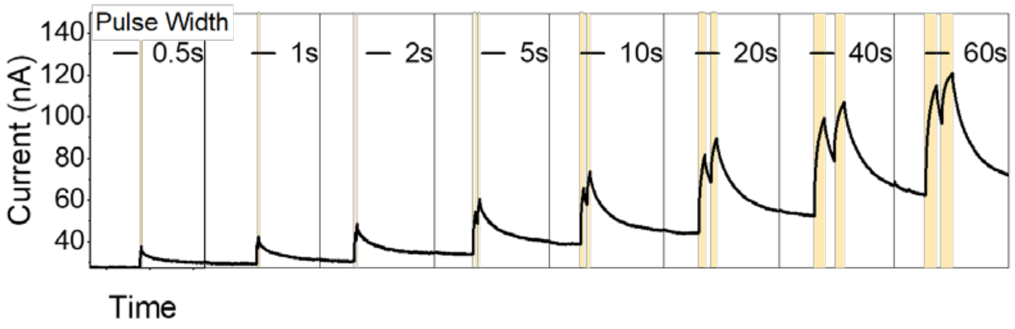

Supplementary Figure 36. Photoelectric responses of the synaptic transistors employing FMM film (DPP-TT) under paired spike with varying pulse width.

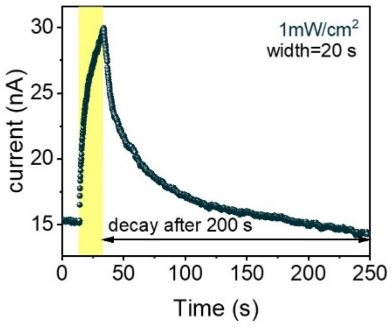

Supplementary Figure 37. EPSC of FMM synapse after 20 s irradiation. The current returned to the original state after 200 s.

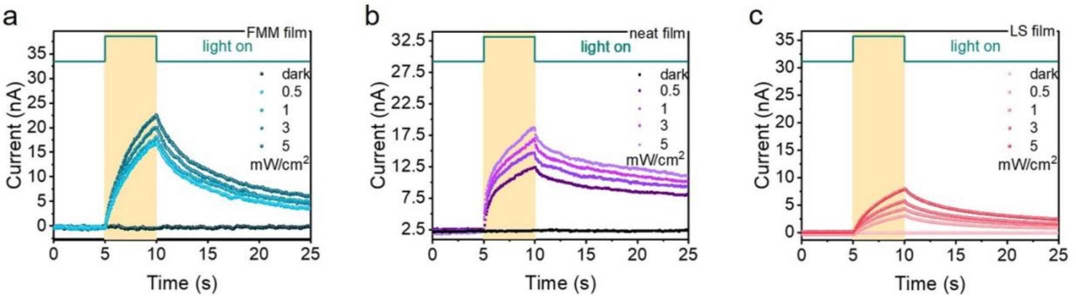

Supplementary Figure 38. Photoelectric responses of the synaptic transistors employing (a) FMM and (b) neat film and (c) LS film of DPP-TT under varying irradiation intensities. Here, we measured the responsibility of the transistors. Responsibility (R) is defined as $R=I_{ph}/P$, where I_{ph} is the difference of drain currents between dark and illuminated states, and P is the incident power. The incident power was scaled by the light intensity and the irradiation area of the device. The light intensity was controlled by the light source, and the irradiation area was defined by the channel length and channel width, which is 5000 µm * 100 µm=5*10^{-3} cm^2.

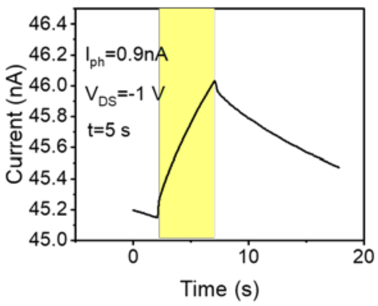

Supplementary Figure 39. EPSC triggered by a single pulse of 5 s at VDS=-1 V. The power of optical pulse was 0.5 mW/cm^2. And the device channel length was 20 µm. A rather low energy consumption of 4.5 nJ was obtained in this device.

Supplementary Table 7. List of previously reported organic synaptic phototransistors with our FMM device.

reference	Semiconducting layer	Stretchability	Consumption (fJ)	V_{DS} (V)	Duration (ms)
[1]	P3HT-b-PPT(5F)/PMMA	Flexible	1.82	-1×10^{-3}	1000
[2]	Pbs QDs/PMMA/Pentacene	Flexible	0.55	-1×10^{-2}	100
[3]	Dif-TES-ADT	Flexible	0.07	-1×10^{-1}	250
[4]	C8-BTBT/P(VDT-TrFE)	Flexible	0.05	-2.5×10^{-5}	20
[5]	DPP-TT	Flexible	0.7×10^{6}	−2	200
[6]	PEDOT:PSS/GOPS	140%	1.98×10^{10}*	−0.6	132

		stretchable			
[7]	SC-SWCTs+CdSe/ZnS QDs	80% stretchable	1.54×10^{-2}	−0.000001	20
[8]	$CsPbBr_3$ QCM film /DPP-TT CONPHINE film	100% stretchable	0.015	-1×10^{-6}	100
[9]	N2200/s-CNT	50% stretchable	4000	1	50
[10]	P3HT NFs	50% stretchable	2.36×10^{9}*	1	50
[11]	PTDPP-Se-6Si	50% stretchable	3.7×10^{-3}*	-1×10^{-4}	10
[12]	DPP-g2T	60% stretchable	1.995×10^{8}*	−0.7	100
[13]	P3HT/PEO NWs	50% stretchable	1.975×10^{14}*	0.5	100
This work	DPP-TT/PBAT FMM film	125% stretchable	4.5×10^{6}	−1	5000

The "*" in table indicated that the energy consumption were not published directly and were derived by the given equations and graphs.

Supplementary Table 8. Comparison of the PPF index of reported stretchable synaptic phototransistors before and after stretching.

reference	Semiconducting layer	PPF index (%)	Δt (ms)	PPF index (%) at stretched states	$\frac{\Delta PPF}{PPF}$ (%)
[14]	IDT-BT	191	500	123 (ε=100%)	35.6
[8]	$CsPbBr_3$ QCM film	148	500	232 (ε=100%)	56.7
[9]	N2200	177	50	157* (ε=50%)	−11.2
[9]	N2200/s-CNT	200	250	2.14 *(ε=50%)	114
[9]	N2200/s-CNT	199	250	1.32 *(ε=50%)	−33.6
[10]	P3HT NFs	146	40	114 *(ε=50%)	−22
[11]	PTDPP-Se-6Si	185	1000	167 *(ε=50%)	−10
[13]	P3HT/PEO NWs	273	50	146 *(ε=50%)	−47
[6]	PEDOT:PSS/GOPS	477	132	380 *(ε=100%)	−20
[7]	SC-SWCTs+CdSe/ZnS QDs	206	400	141 *(ε=80%)	−32
This work	DPP-TT/PBAT FMM film	130	1000	138 (ε=100%)	6.7

The "*" in table indicated that the PPF index were not published directly and were derived by the given equations and graphs.

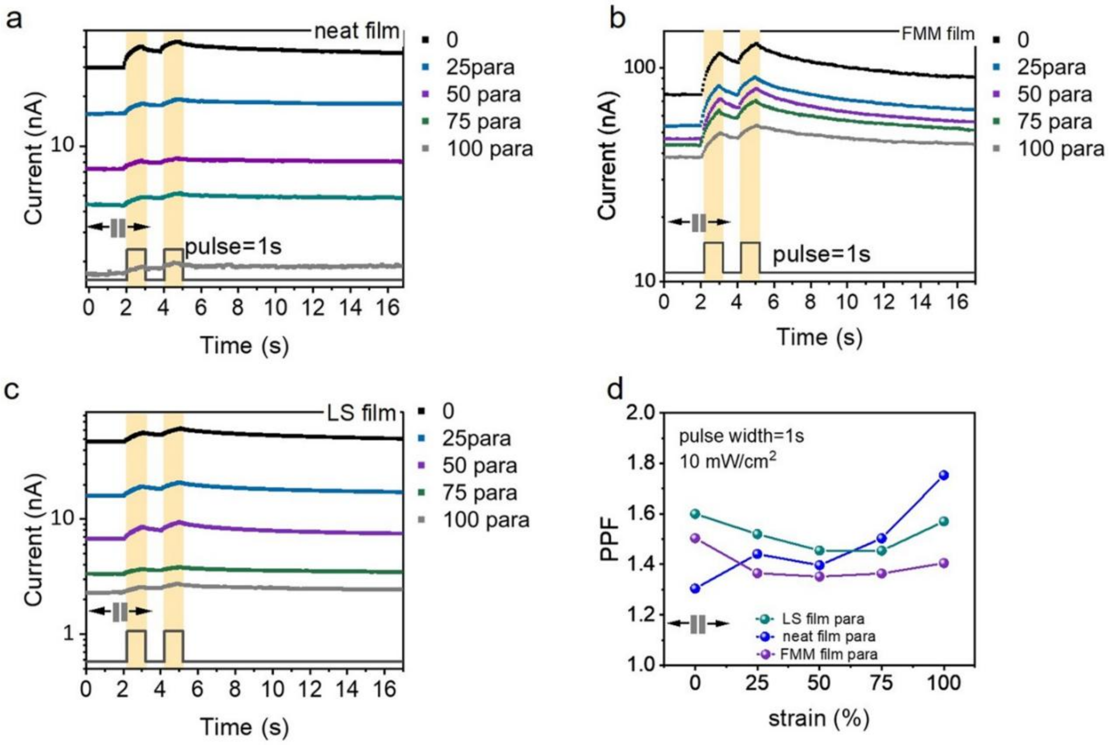

Supplementary Figure 40. EPSC under paired pulses of the stretchable synaptic phototransistors under strain. (a) Synaptic transistor employing neat film, (b) Synaptic transistor employing FMM film, (c) Synaptic transistor employing LS film. (d) PPF index of the stretchable synaptic transistors under strain. The irradiation power was 10 mW cm^{-2}. The wavelength was 760 nm.

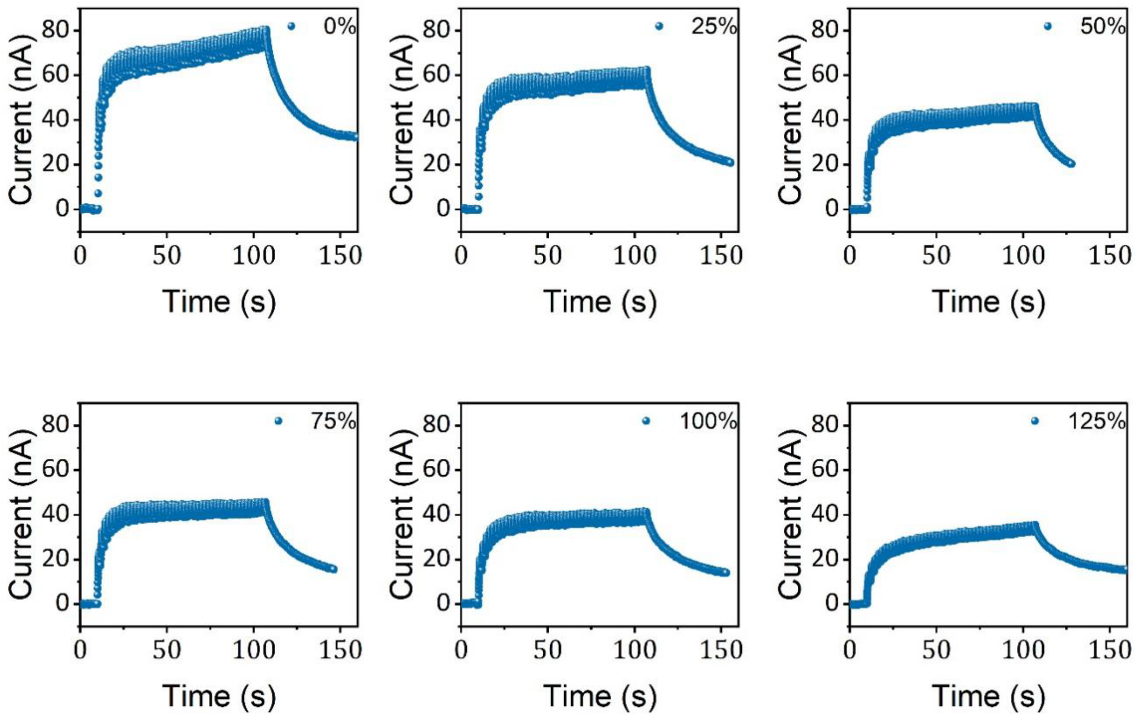

Supplementary Figure 41. Strain-tolerance long-term response of the synaptic phototransistors employing FMM film upon 50 continuous pulses with a width of 1s. Strain was parallel to the charge transport.

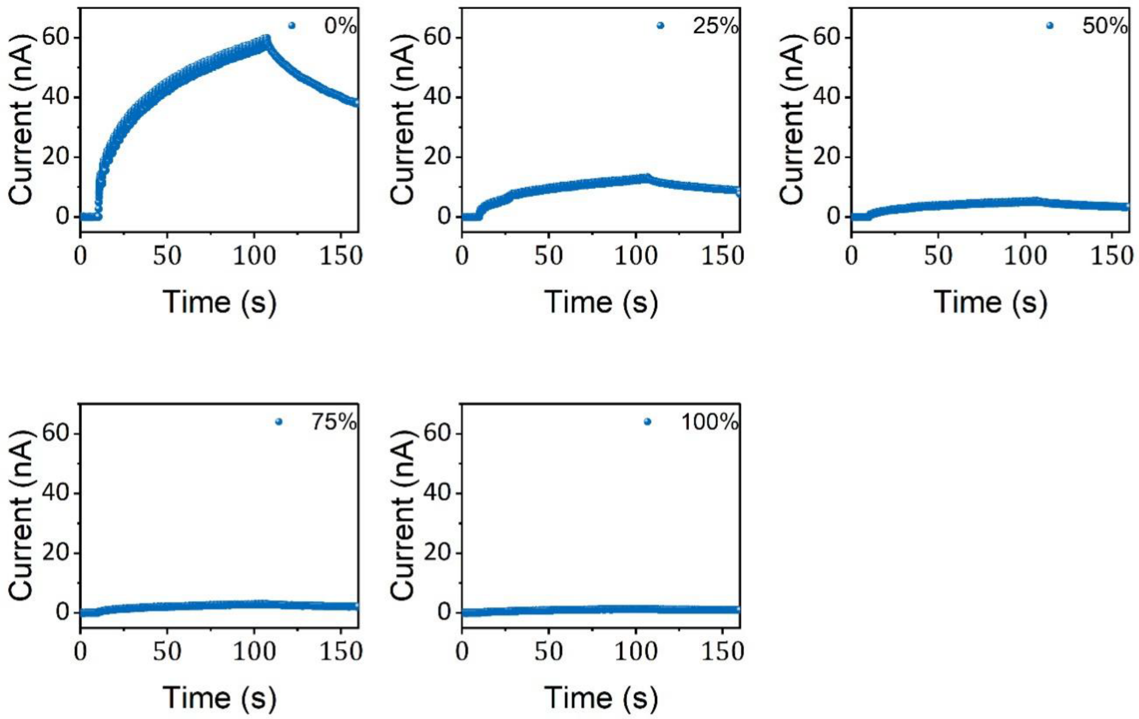

Supplementary Figure 42. Strain-tolerance long-term response of the synaptic phototransistors employing neat film (DPP-TT) upon 50 continuous pulses with a width of 1s. Strain was parallel to the charge transport.

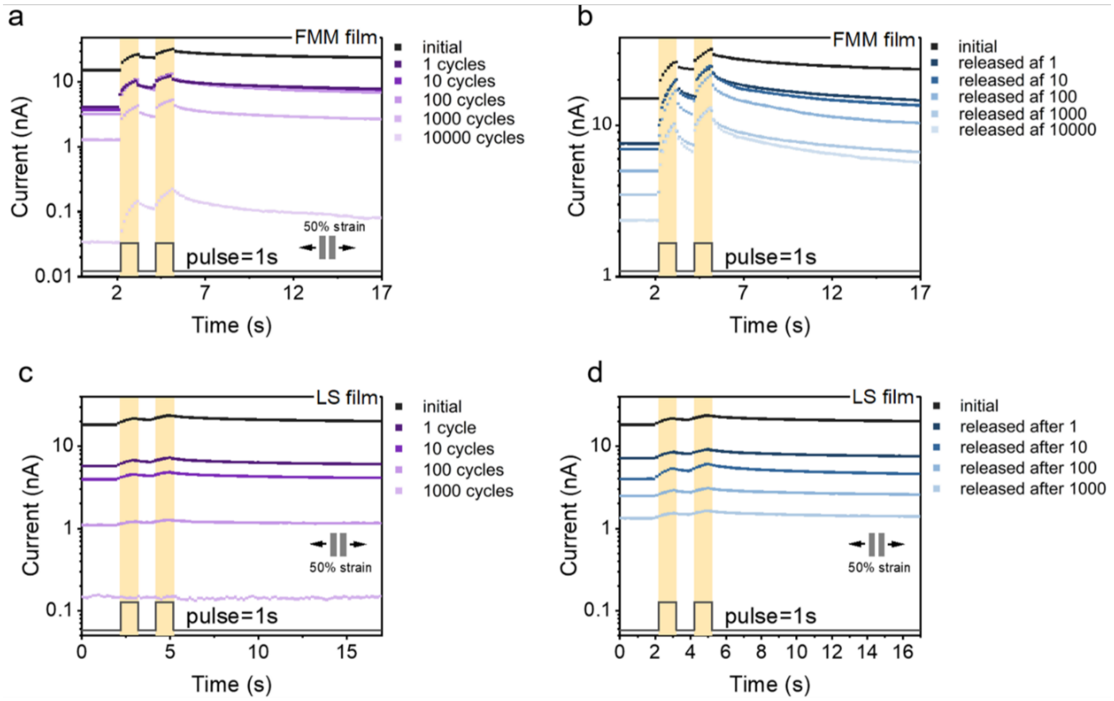

Supplementary Figure 43. Photoelectronic stability of the synaptic phototransistors employing FMM film and LS film of DPP-TT upon repeating stretching-releasing cycles (up to 10000 cycles) at 50% strain. The irritation intensity was 1 mW cm^{-2}.

Supplementary Notes

1. Finite element analysis (FEA) of the porous film under uniaxial tensile stress

Assume that the conjugated polymers are homogeneously dispersed in the plastic polymer matrix and occupy each single unit. Two samples are assigned the same material properties and film thickness ($d \ll W = 1/2L$, d, W and L are the thickness, the width and the length of the films, respectively), but different pore diameters that differed by a factor of ten (for P1, R1=3*d*, a representative of the red zone, for P2, R2=30*d*, a representative of the blue zone). Here, the Young's modulus of both films is taken from previous work, M= 340 MPa[15].

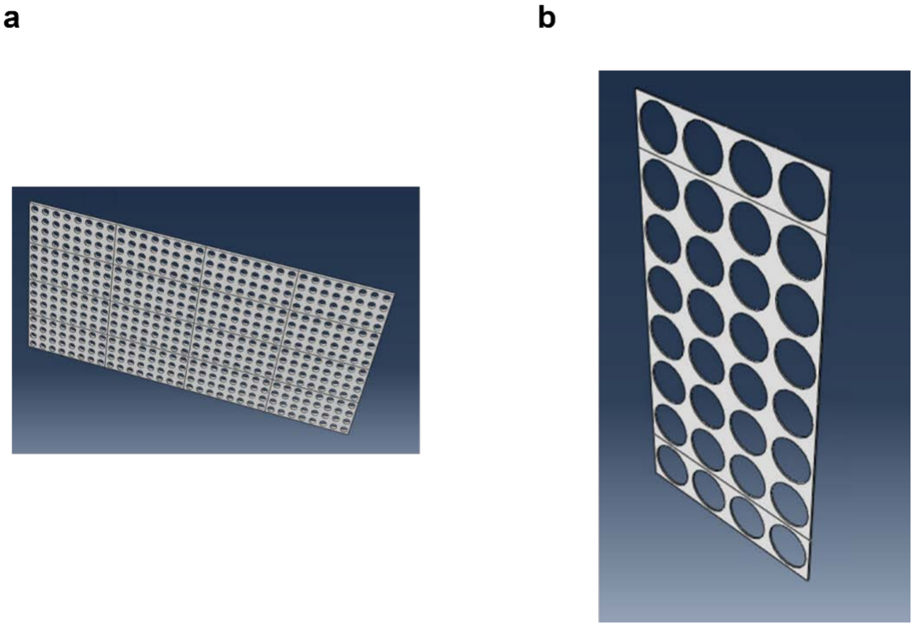

Supplementary Figure 44. The models at initial status. a. P1**,** b. P2

2. Abbreviations

AFM: atomic force microscope
CP: conjugated polymer
CP/SEBS: composite of conjugated polymer (CP) and SEBS
COS: crack-onset strain
CT-inverter: Complementary inverter
DMT modulus: Derjaguin–Muller–Toporov modulus
DPP-g2T: poly(2,5-bis(2-octyldodecyl)-3,6-di(thiophen-2-yl)-2,5-diketopyrrolopyrrole-alt-2,5-bis(3-triethyleneglycoloxythiophen-2-yl))
DPP-TT: Thieno[3,2-b]thiophene-diketopyrrolopyrrole
EPSC: Excitatory Postsynaptic Current
FEA: Finite element analysis
FMM: Fibrous micromesh
GIWAXS: Grazing incidence wide angle X-ray scattering

Insul.: insulator
L-L phase separation: liquid–liquid phase separation
L-S separation: liquid–solid separation
LS: Lateral phase separation
LTP: Long-Term Plasticity
N2200: Poly{[N,N′-bis(2-octyldodecyl)-naphthalene-1,4,5,8-bis(dicarboximide)-2,6-diyl]-alt-5,5′-(2,2′-bisthiophene)}
N2200-SEBS: Hybrid film of N2200 and SEBS
OFET: organic field-effect transistor
OM: Optical microscope
OSC: organic semiconductor
P3HT: Poly(3-hexylthiophene-2,5-diyl)
PBAT: Poly(butylene adipate-co-terephthalate)
PDMS: Polydimethylsiloxane
PPF: Paired-Pulse Facilitation
PU/AgNW: composite of polyurethane rubber (PU) and Ag nanowires (AgNW)
SEBS: Styrene–Ethylene–Butylene–Styrene
SEM: scan electron microscope
STP: Short-Term Plasticity
UV-vis: ultraviolet–visible

Supplementary References

1. Jiang, L. *et al.* One-step preparation of semiconductor/dielectric bilayer structures for the simulation of flexible bionic photonic synapses. *ACS Appl. Mater. & Interfaces* **15**, 7227–7235 (2023).
2. Zhang, J. *et al.* Retina-inspired artificial synapses with ultraviolet to near-infrared broadband responses for energy-efficient neuromorphic visual systems. *Adv. Funct. Mater.* **33**, 2302885 (2023).
3. Shi, J. *et al.* A fully solution-printed photosynaptic transistor array with ultralow energy consumption for artificial-vision neural networks. *Adv. Mater.* **34**, 2200380 (2022).
4. Li, Q. *et al.* Ultralow power wearable organic ferroelectric device for optoelectronic

neuromorphic computing. *Nano Lett.* **22**, 6435–6443 (2022).

5. Liang, Z. *et al.* Organic optoelectronic synaptic transistor with enhanced UV light response based on insulating polymer-assisted p–n heterojunction. *ACS Appl. Mater. & Interfaces* **16**, 65091–65099 (2024).
6. Matsuhisa, N. *et al.* High-transconductance stretchable transistors achieved by controlled gold microcrack morphology. *Adv. Electron. Mater.* **5**, 1900347 (2019).
7. Xie, T. *et al.* Carbon nanotube optoelectronic synapse transistor arrays with ultra-low power consumption for stretchable neuromorphic vision systems. *Adv. Funct. Mater.* **33**, 2303970 (2023).
8. Wang, C. *et al.* Strain-insensitive viscoelastic perovskite film for intrinsically stretchable neuromorphic vision-adaptive transistors. *Nat. Commun.* **15**, 3123 (2024).
9. Shim, H. *et al.* An elastic and reconfigurable synaptic transistor based on a stretchable bilayer semiconductor. *Nat. Electron.* **5**, 660–671 (2022).
10. Shim, H. *et al.* Stretchable elastic synaptic transistors for neurologically integrated soft engineering systems. *Sci. Adv.* **5**, eaax4961 (2019).
11. Lan, L. *et al.* Stretchable optoelectronic synapses with ultraviolet to near-infrared perception for retina-inspired computing and vision-adaptive sensing. *NPJ Flex. Electron.* **9**, 16 (2025).
12. Chen, J. *et al.* Highly stretchable organic electrochemical transistors with strain-resistant performance. *Nat. Mater.* **21**, 564–571 (2022).
13. Liu, L. *et al.* Stretchable neuromorphic transistor that combines multisensing and

information processing for epidermal gesture recognition. *ACS nano* **16**, 2282–2291 (2022).

14. Xu, F. *et al.* Intrinsically stretchable photonic synaptic transistors for retina-like visual image systems. *J. of Mater. Chem. C* **10**, 10586–10594 (2022).
15. Zokaei, S. *et al.* Tuning of the elastic modulus of a soft polythiophene through molecular doping. *Mater. Horiz.* **9**, 433–443 (2022).
